# How to Combat the Global Opioid Crisis

**Published:** 2023-01-23

**Authors:** Catherine Dennen A., Kenneth Blum, Eric Braverman R., Abdalla Bowirrat, Marks Gold, Igor Elman, Panayotis Thanos K., David Baron, Ashim Gupta, Drew Edwards, Rajendra Badgaiyan D.

**Affiliations:** 1 Department of Family Medicine, Jefferson Health Northeast, Philadelphia, PA., USA; 2 The Kenneth Blum Behavioral & Neurogenetic Institute, LLC., Austin, TX 78701, USA; 3 Center for Sports, Exercise, Psychiatry, Western University Health Sciences, Pomona, CA., USA; 4 Institute of Psychology, ELTE Eötvös Loránd University, Budapest, Hungary; 5 Department of Molecular Biology and Adelson School of Medicine, Ariel University, Ariel, Israel; 6 Department of Psychiatry, Washington University School of Medicine, St. Louis, MO., USA; 7 Cambridge Health Alliance, Harvard Medical School, Cambridge, MA., USA; 8 Behavioral Neuropharmacology and Neuroimaging Laboratory, Department of Pharmacology and Toxicology, Jacobs School of Medicine and Biomedical Sciences, Clinical Research Institute on Addictions, University at Buffalo, Buffalo, NY., USA; 9 Department of Psychology, University at Buffalo, Buffalo, NY., USA; 10 Future Biologics, Lawrenceville, GA., USA; 11 Drew Edwards & Associates, Lakeview, FL, USA; 12 Department of Psychiatry, South Texas Veteran Health Care System, Audie L. Murphy Memorial VA Hospital, Long School of Medicine, University of Texas Medical Center, San Antonio, TX 78229, USA

**Keywords:** Opioid Use Disorder (OUD), Substance Use Disorder (SUD), Genetic Addiction Risk Severity (GARS), Preaddiction, Brain Health Check (BHC), Drug Overdoses

## Abstract

Since 2000 there have been 915,515 people who have died from a drug overdose in the United States (US). This number continues to increase and in 2021 drug overdose deaths reached a record high of 107,622, and opioids specifically were responsible for 80,816 of those deaths. This unprecedented rate of drug overdose deaths is the direct result of increasing rates of illicit drug use in the US. It was estimated that in the US in 2020, approximately 59.3 million individuals had used illicit drugs, 40.3 million had a substance use disorder (SUD), and 2.7 million had opioid use disorder (OUD). Typical treatment for OUD involves an opioid agonist (i.e., buprenorphine or methadone) along with a variety of psychotherapeutic interventions (i.e., motivational interviewing, cognitive-behavioral therapy (CBT), behavioral family counseling, mutual help groups, etc.). In addition to the aforementioned treatment options, there is an urgent need for new therapies and screening methods that are reliable, safe, and effective. Similar to the concept of prediabetes is the novel concept of “preaddiction.” Preaddiction is defined as individuals with mild to moderate SUD or those at risk for developing a severe SUD/addiction. Screening for preaddiction could be achieved through genetic testing (i.e., the genetic addiction risk severity (GARS) test) and/or through other neuropsychiatric testing (i.e., Memory (CNSVS), Attention (TOVA), Neuropsychiatric (MCMI-III), Neurological Imaging (qEEG/P300/EP)). The concept of preaddiction, when used in conjunction with standardized and objective diagnostic screening/testing, would halt the rise of SUD and overdoses with early detection and treatment.

## Introduction

### Epidemiology

From 2000–2020 there have been 915,515 people who have died from a drug overdose in the United States (US) [1,2]. This number has been steadily rising since 2018 and according to provisional data from the Centers for Disease Control and Prevention’s (CDC) National Center for Health Statistics in 2021, reached record highs with an estimated 107,622 overdose deaths (Table 1) [3]. In addition, the majority of the overdose deaths were related to opioids. Currently, the US has the world’s highest number of opioid-involved deaths per capita. Specifically, opioids were responsible for approximately 80,816 of the 107,622 deaths in 2021, which is also an approximate 15% increase when compared to the 70,029 opioid related overdose deaths in 2020 [3].

This unprecedented rate of drug overdose deaths is the direct result of increasing rates of illicit drug use in the US. According to data from the Substance Abuse and Mental Health Services Administration’s (SAMHSA) National Survey on Drug Abuse and Health (NSDUH), illicit drug use has been steadily increasing since 2015 (Table 2) [3,4]. In particular, it was estimated that in the US in 2020, approximately 21.4% (59.3 million) of individuals had used illicit drugs and 40.3 million individuals had a substance use disorder (SUD) [4].

As previously mentioned, opioid addiction and opioid use disorder (OUD) continue to be epidemics in the US and around the world. According to SAMHSA, in 2020 approximately 9.5 million individuals in the US, aged 12 or older, will have misused opioids [4]. Prescription pain relievers were found to be the most commonly misused opioids, and out of the 9.5 million individuals who had misused opioids, 9.3 million had misused prescription pain relievers, compared to 902,000 individuals who had used heroin [4]. In addition, approximately 2.7 million people in the US, aged 12 or older, had an opioid use disorder (OUD) in 2020 [4]. Worldwide, it is estimated that 16 million individuals currently have OUD or have suffered from it in the past [5].

The majority of individuals with SUD/OUD will need some form of treatment. In 2020, 41.1 million (14.9%) individuals in the US aged 12 and older needed treatment for their substance use [4]. The need for substance uses treatment has more than doubled since 2017, when only approximately 20.7 million individuals in the US, aged 12 and older, required treatment for their substance use [4]. However, only 2.6 million (6.5%) individuals, aged 12 or older, received treatment for their substance use in 2020 [4].

Treatments for addiction, specifically opioids, are outdated, have limited efficacy, poor adherence, and high relapse rates [6]. However, research has shown that medication for OUD (MOUD) improves treatment retention, promotes abstinence, and has been linked to a decrease in mortality [7–11]. The initial treatment course for individuals that suffer from moderate or severe OUD involves an opioid agonist, such as buprenorphine or methadone, along with adjunctive psychosocial interventions. Buprenorphine is typically preferred over methadone because of its greater accessibility, lower risk of death with overdose, and fewer drug-drug interactions [12,13]. However, for individuals with greater levels of tolerance and physical dependence methadone is the preferred agent. An acceptable alternative for those who refuse or are unable to receive agonist treatment is the opioid antagonist naltrexone. Additionally, our laboratory has studied the use of naltrexone combined with a pro-dopamine regulator (KB220) to establish a transient balance of dopamine function throughout the brain reward circuitry [14,15]. However, individuals must go through medically supervised withdrawal before starting naltrexone. The best outcomes have been seen with prolonged medication-assisted treatment (MAT) therapy, but there have been concerns related to the effects of prolonged opioid substation therapy [16]. Finally, the short-acting opioid antagonist naloxone is the drug of choice for treating opioid overdoses.

Currently, in the US and in many other countries across the world, treatment for OUD involves simply substituting one addiction for a similar chemical addiction [17]. Recent data suggests that overdose risk continues long after patients’ complete treatment with buprenorphine [18]. This is not surprising given that many people with OUD have well-known polymorphic risk alleles that increase the risk of addictive-like behaviors, including OUD [19]. Discontinuation of MAT is associated with high rates of relapse and an increased risk of overdose [20]. In addition, similar to treatment-resistant depression, there is a subpopulation that does not respond to standard OUD treatments, including MAT. Patients who have treatment-resistant opioid use disorder (TROUD) and/or relapse are often given the same MATs again and again and again [21]. The risk of overdose and relapse remains even after years of stable recovery. Recovery can be a “remission” of the symptoms of an OUD but not its elimination [22]. There has been incremental progress in understanding the genetic [23], epigenetic [24], onset [25], route [26], lived experiences, and other risks in developing OUDs. It is now understood that the best workaround is to reduce harm by reducing risks to the user, reversing overdose deaths, and initiating opioid replacement for an extended period of time as if the user had an opioid deficiency syndrome [27].

A variety of psychotherapeutic interventions can be utilized either alone or in conjunction with MAT to treat OUD/SUD, including motivational interviewing, cognitive-behavioral therapy (CBT), behavioral family counseling, and mutual help groups. Motivational interviewing is a psychotherapeutic technique designed to help individuals identify and overcome ambivalence to behavior change. It can be used to treat individuals with OUD who are overtly ambivalent or struggling to cease their substance use and has been found to be effective in the treatment of SUD [28–31] and as an adjunct to MOUD[32,33]. CBT is another technique that is based on the idea that emotions, thoughts, and tactile sensations influence behaviors, such as opioid use. CBT can help individuals with OUD in becoming aware of their cognitive distortions that have a detrimental effect on their mood and propensity for relapse, and in the development of a set of skills to accept or modify these cognitive distortions. Several studies have shown the effectiveness of CBT in the treatment of SUD, OUD, and other addictive behaviors [34–38]. Behavioral family counseling has also been found to increase treatment compliance and substance use disorder outcomes in individuals with OUD [39,40]. Mutual help groups such as methadone anonymous, narcotics anonymous, and medication-assisted recovery services can also be used to provide help and support to individuals struggling with OUD. Studies have shown that these groups are effective and help improve the quality of life among individuals with OUD/SUD [41–44]. However, due to the anonymity of these programs research is limited.

In addition to the aforementioned treatment options, there is an urgent need for new therapies and screening methods that are reliable, safe, and effective. Despite decades of federal funding for the research, development, and implementation of innovative therapies for those with serious addictions, treatment penetration rates have been found to be less than 20% [45,46]. The diabetes field faced a similar situation but were able to increase treatment penetration and impact by identifying those at risk for diabetes and intervening during the early stages of the disease, which became known as prediabetes [47]. The concept of prediabetes has resulted in an increased efficiency of early detection, decreased time between symptom onset and initiation of treatment, and has been successful in slowing the progression of diabetes [48]. Similar to the concept of prediabetes is the novel concept of “preaddiction,” which has been suggested for inclusion in the DSM. Preaddiction is defined as individuals with mild to moderate SUD or those at risk for developing a severe SUD/addiction. While prediabetes is considered a manifestation of failing homeostatic function, preaddiction may be closely related to [49] hedonistic derailments [50], namely, hypodopaminergia in the meso-limbic brain reward circuitry [51], as well as the associated opioidergic-, serotonergic-, cannabinergic, GABA-ergic, glutaminergic, and cholinergic abnormalities and clinical manifestations [52,53]. Screening for preaddiction could be achieved through genetic testing. Specifically, the genetic addiction risk severity (GARS) test is as a genetic predictor of risk that was initially developed to identify those at risk for alcohol use disorder (AUD) and other reward deficiency syndrome (RDS) behaviors (i.e., addictive, impulsive, and compulsive behaviors), including OUD [54–59]. The GARS test consists of ten genes and eleven associated risk alleles. In addition, when GARS scores are coupled with the Addiction Severity Index (ASI)-media version 5 they were found to be able to significantly predict drug and alcohol severity, respectively [55,56]. Currently, despite the existence of the GARS test and possibly other viable genetically based panels, most clinicians fail to identify individuals with SUD/OUD appropriately and objectively. This problem is the result of a lack of psychometrics for assessing the premorbid risks of SUD in the primary care setting [60].

SUD is widely accepted as a neurological disorder that can affect memory, attention, and mental health. Despite this, the amount of routine, standard, and objective assessments being performed to evaluate brain health in the primary care setting is extremely limited (i.e., the mini-mental status exam (MMSE), patient health questionnaire-9 (PHQ-9), and the Conners Comprehensive Behavior Rating Scale (CBRS), etc.). A standardization for how we approach brain health/addiction is vital to halting the addiction pandemic. For example, it took roughly 40 years for the medical community to establish a standard, routine set of cardiac tests (i.e., blood work, electrocardiogram (EKG), echocardiogram, etc.), that ultimately halted the bypass cardiac pandemic. Brain health needs to have a similar stepwise approach implemented by the entire medical community. A study by Braverman et. al., attempts to provide this standardization by establishing a “Brain Health Check,” which is based on precision medicine for the SUD phenotype and centered around precision neuropsychiatric testing, i.e., Memory (CNSVS), Attention (TOVA), Neuropsychiatric (MCMI-III), Neurological Imaging (qEEG/P300/EP) [61]. The Brain Health Check examines and establishes a set of objective assessments that parallel those used to screen for and treat cardiac disease (Table 3) [61]. This approach, or one similar, would greatly improve brain/mental health and halt the rise of SUD and overdoses with early detection and treatment.

## Conclusion

In conclusion, there is an urgent need for new therapies and screening methods that are reliable, safe, and effective. In addition, a standardized approach for how we approach addiction is necessary. Similar to the concept of prediabetes is the novel concept of “preaddiction.” Screening for preaddiction could be achieved through genetic testing (i.e. the genetic addiction risk severity (GARS) test) and/or through other neuropsychiatric testing (i.e., Memory (CNSVS), Attention (TOVA), Neuropsychiatric (MCMI-III), Neurological Imaging (qEEG/P300/EP)). The concept of preaddiction, when used in conjunction with standardized and objective diagnostic screening/testing, would greatly improve brain/mental health and stop/prevent the rise of SUD and overdoses with early detection and treatment.

## Figures and Tables

**Table 1:** Drug Overdose Deaths in the United States [1]

2018	2019	2020	2021
67,367	70,630	91799	107,622

**Table 2:** Illicit Drug Use: Among People Aged 12 or Older [4]

2015	2016	2017	2018	2019	2020
17.8%	18.0%	19.0%	19.4%	20.8%	21.4%

**Table 3:** Parallel pattern to manage brain and cardiac disease^1^

Core Brain Domains/Tests	Core Cardiac Domains/Tests
Memory: CNSVS	Blood pressure
Attention: TOVA	Blood work: cholesterol, CRP, etc.
Neuropsychiatric: MCMI-III	Electrophysiology: EKG
Genetic: GARS	Echocardiogram: Valves, Ejection fraction
Imaging: Electrophysiology, qEEG/p300/EP	Imaging: CT angiogram
Supplemental Brain Testing	Supplemental Cardiac Testing
Memory: WMS, MCI Screening, MMSE	Electrophysiological: Holter, EP testing
Attention: Connors, ADD Checklist	Echocardiogram: Transesophageal
Neuropsychiatric: TT, MBTI, MMPI	Blood work: BNP, cardiac enzymes, etc.
Imaging: MRI, PET, SPECT	Imaging: Catheterization, MRI, PET

1 CNSVS: Central Nervous System Vital Signs, TOVA: Test of Variables of Attention, MCMI-III: Millon Clinical Multiaxial Inventory III, GARS: Genetic Addiction Risk Score, qEEG: Quantitative Electroencephalogram, EP: Evoked Potential, WMS: Wechsler Memory Scale, MCI: Mild Cognitive Impairment, MMSE: Mini-Mental State Examination, ADD: Attention Deficit Disorder, TT: Type and Temperament, MBTI: Myers–Briggs Type Indicator, MMPI: Minnesota Multiphasic Personality Inventory, MRI: Magnetic Resonance Imaging, PET: Positron Emission Tomography, SPECT: Single-Photon Emission Computed Tomography, CRP: C-Reactive Protein, BNP: B-type Natriuretic Peptide. (Used with permission [61])
